# Effect of enhanced reminders on postnatal clinic attendance in Addis Ababa, Ethiopia: a cluster randomized controlled trial

**DOI:** 10.1080/16549716.2019.1609297

**Published:** 2019-05-24

**Authors:** Abraham Sahilemichael Kebede, IkeOluwapo O. Ajayi, Ayodele O. Arowojolu

**Affiliations:** a Pan Africa University Life and Earth Sciences Institute (Including Health and Agriculture), University of Ibadan, Ibadan, Nigeria; b Department of Epidemiology and Medical Statistics, Faculty of Public Health, College of Medicine, University of Ibadan, Ibadan, Nigeria; c Department of Obstetrics and Gynecology, Faculty of Clinical Medicine, College of Medicine, University of Ibadan, Ibadan, Nigeria

**Keywords:** Appointments reminders, short message service, voice call, postnatal compliance, cluster randomized trial, mobile phones

## Abstract

**Background**: Failure to attend maternal health services is an intractable challenge for the health-care system in low- and middle-income countries. The use of technology for reminding patients about their appointments has been demonstrated to be an effective (future) tool toward increased health care services utilization in developing countries, such as Ethiopia.

**Objective**: We aimed to investigate the effect of enhanced reminders on postnatal care attendance versus usual care (notification of an appointment at discharge).

**Methods**: The study was a cluster randomized controlled trial: out of eligible 86 health centers, 16 health centers in Addis Ababa (AA) were randomized to either the intervention (8) or the control (8) groups; with a total of 350 mothers equally randomized into each arm. Mothers in the intervention group received the SMS (short message service) or a voice call reminder at 48 and 24 hours before the due postnatal appointment, whereas the control group received only the usual notification of appointments provided by health professionals at discharge from the ward following delivery. We recruited participants on wards after delivery at discharge and followed them up to 6 weeks. This study’s primary outcome was postnatal visit compliance. Our assessment consisted of a two-level bivariate and a multivariate ordinal logistic regression analysis.

**Results**: The majority (97.7%) of the participants completed the study; 173(98.9%) of women in the intervention group and 169 (96.5%) of women in the control group. There was a statistically significant difference in postnatal care (PNC) compliance among women who were in the intervention versus the control group (p-value = 0.005). Higher odds of postnatal compliance was observed among the intervention group (AOR:2.98, 95% CI 1.51–5.8).

**Conclusions**: Mobile phone reminders were effective in terms of enhancing adherence to PNC appointments. This indicates integration of mobile phone reminders in postnatal care could improve postnatal appointment compliance.

## Background

According to World Health Organization (WHO) 2016, globally, an estimated 303,000 women died of pregnancy and childbirth-related causes and 2.7 million babies died during the first 28 days of life [1–3]. Provision of quality health care during pregnancy, childbirth and after delivery can prevent many of these deaths, yet globally only 64% of women received recommended four or more antenatal care throughout their pregnancy and only 28% of them received postnatal care [4]. Evidences from WHO indicated that 90% coverage of routine Postnatal Care (PNC) and curative care will avert 10 to 27% of newborn death, which imply high PNC coverage could save up to 310,000 newborn lives per year in Africa [5,6].

**Figure 1.**
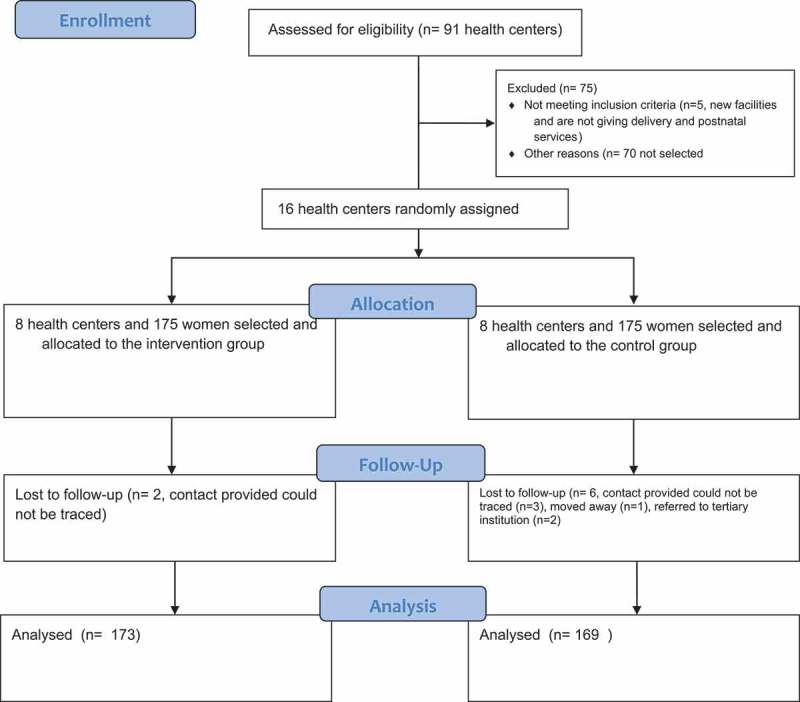
Flow diagram of trial randomization, allocation, follow up and analysis.

**Figure 2.**
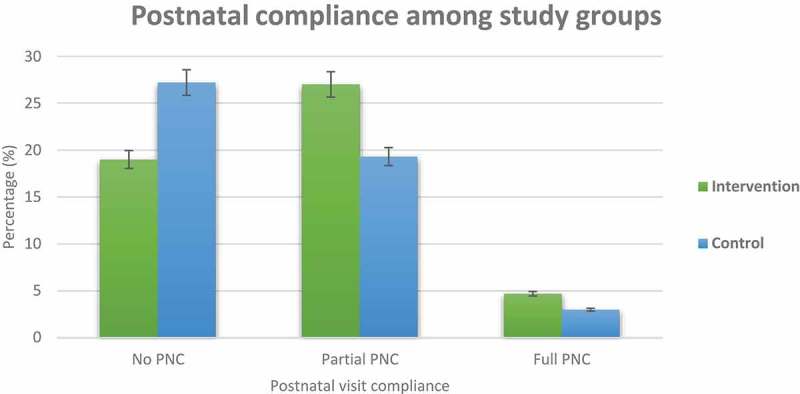
Proportion of postnatal visit compliance among the intervention and control groups.

Despite the higher emphasis on safe motherhood initiative in ensuring the accessibility and utilization of postnatal services, it is still one of the neglected care point in the continuum of maternal care [7,8]. Rural residency, mother’s educational status, number of ANC visits, place of delivery, perceived distance from the health facility and past postnatal experience were among the reasons mentioned for the low coverage of PNC service [9–12].

Failure to attend maternal health services are an intractable challenge to the health-care system in low and middle-income countries [13]. Missed appointment have caused inefficiency in the health-care system due to the cost generated by non-attendance (underutilization of equipment and personnel) and delayed diagnosis and treatment, which might have contributed to the higher maternal morbidity and mortality [13–15].

Nowadays, fast-growing mobile network coverage is sought as one of the opportunities to improve the missed appointments from health service care [16–19]. Worldwide mobile phone coverage has increased by 62% since 2002 with expected more than 80% coverage in the year 2020 [19]. In sub-Saharan Africa (SSA) alone in 2010, more than 70% of the population had mobile phone access which exceeded other services like access to improved water and improved sanitation facilities which remained 61% and 31%, respectively [20].

In the last 10 years growing evidence showed that mobile phone reminders (text messages and phone calls) can be used to improve attendance to health-care appointments, increase access to health care, improve adherence to treatment, follow-up and rehabilitation and improves the efficiency of health service delivery [21–23]. Randomized trials indicated mobile phone reminder improves appointment compliance [20,23,24], adherence to ART and Tb medications [25,26]. In addition, it facilitates provision of health education, counseling [27,28] and behavioral change interventions [17,29].

In Ethiopia, mobile phone technology access is expanding rapidly [30]. The 2011 vital wave report indicated, the mobile phone coverage in Ethiopia was as high as 85% [31,32]. Despite this promising and fast-growing technology/area, there are few evidences on the possible role of mobile health on different health services in Ethiopia. In addition, studies suggested that the reasons for low postnatal care utilization in Ethiopia include forgetfulness and discharge without any indication and appointments on when and where to seek further care or support [33]. In this paper, we aimed to assess the effect of enhanced mobile phone reminders and their acceptability on postnatal care uptake among women who gave birth in public health facilities in Addis Ababa.

## Methods

### Study settings

This study was conducted in Addis Ababa, the capital city of Ethiopia. The city is divided into 10 administrative sub-cities and 116 kebeles (the smallest administration unit in Ethiopia). In 2016, the total population of Addis Ababa was estimated to be 4 million with a population annual growth rate of nearly 2.9% per year [34]. According to Addis Ababa Health Bureau (AAHB) report, there are 91 health centers in AA. These health centers are primarily responsible for the provision of primary health care (PHC) services including ANC, delivery and postnatal care services. In the Ethiopian health-care system, health centers have a specific catchment populations and area to serve. More than 90% of the mothers access delivery and postnatal services in the health center serving the catchment population, while the rest gets a referral to higher health facilities [35]. The mobile phone subscription rate in AA is more than 80% making it ideal for mobile health (mHealth) interventions [36].

### Study design and participants

A cluster randomized control trial was conducted in 16 (eight matched pairs) health centers (eight interventions; eight control) across Addis Ababa, Ethiopia between August and November, 2017. The study recruited participants at wards after delivery and followed them for 6 weeks at postnatal clinics.

There were 10 sub-cities in AA and they were stratified into two strata (stratum 1 = 6 sub cities and stratum 2 = 4 sub cities) based on geographical location, the size of geographical areas, population density, and economic activities. Two sub-cities were selected from each stratum using simple random sampling. All health centers found in the selected sub-cities of Addis Ababa were eligible to be included in this study. Health centers were excluded if they do not give maternal care (ANC, delivery and postnatal care). All women who gave birth in selected public health centers in Addis Ababa within the study period were eligible for inclusion. Mothers were counseled for participation in the study and provided with all information required. They were recruited if they owned or had regular access to phones, and gave written informed consent for participation in the study. Mothers were excluded if they were having serious obstetrical and/or medical complications requiring hospitalization during the postnatal period or beyond the study period, and were unwilling to participate in the study. Women who indicated that they planned to receive postnatal care outside the intended health center and those who had provided incorrect contact information such that they could not be traced were excluded from this study.

### Randomization and blinding

Before the randomization process began health centers in selected sub cities were assessed for eligibility (5 health centers were excluded because they don’t give the service). An independent intervention manager who was unaware of the objective of the study randomized the health centers (1:1) to the mobile reminder intervention or usual care using a computer-generated randomization sequence. The health centers were randomized in matched pairs and this was done based on the geographic non-adjacency which was extracted from health facilities distribution in AA. Within each matched pair, health centers were randomly assigned to either intervention or control groups there and after. The data collectors were not masked to the intervention but were asked not to inform assessors (research assistants responsible for the end line data collection). Figure 1 shows the enrollment and lost to follow-up according to the consort extension 2010 for cluster randomized trial.

The number of women to be included from each health center was proportionately allocated based on three-month average delivery flow. All mothers who gave birth in the selected health centers and met the inclusion criteria were recruited consecutively into the study.

### Sample size determination

The sample size was determined using two population proportion formula for cluster randomized trial [37,38]. The sample size calculation assumed α (level of significance) = 1.96); β (power) = 80% (0.845); the baseline rate of postnatal visit in Addis Ababa = 55% [39] and an increase of 21% expected based on a quasi-experimental study done in Nigeria comparing SMS reminder intervention with a historical control for postnatal care attendance [40]. The intervention to control ratio was 1:1; lost to follow-up of 10% and the design effect of 2 was used to estimate the minimum sample size of n = 175 in each group.

### Interventions

We used open-sourcing software, frontline SMS version 2.6.5 (Occam Technologies Inc.) to manage the schedule and send short messages to participating mothers in the intervention group. The software works in combination with internet, a computer, and android phone. After installation, the SMS was managed using frontline sync. The scheduler and the open-sourcing SMS software were downloaded and refreshed daily, and connected to broadband internet by the intervention managers. According to the Ethiopian national guideline for the postnatal care at least three visits on the 3rd, 7th and between 8th and 42nd days are recommended. Women who came to health centers in the intervention arm received a sequential reminder (SMS or Voice) 48 and 24 hours before their next appointment.

The content of reminders was prepared based on literature search and consultations with communication expert. A SMS reminder content of less than 164 characters was designed validated and pretested to check whether the message was consistently understood by the receivers. The message was prepared in English and translated to local language (Amharic) version; thereafter back translation to English was done to verify accuracy of the translation by an expert in both English and the local language. After recruitment of the study participants the data collectors sent the details of the mother including contact number, preferred mode of reminder, date of delivery and details of the health facility to the intervention manager. The intervention manager was a nurse trained by the principal investigator on how to make a voice call, update and refresh the database. The intervention manager was in charge of making calls and ensuring that the SMS were sent according to the schedules. We used the confirmatory mode to verify whether the messages were sent or delivered to study participants. If the messages were not delivered multiple SMS and voice calls were tried. However, the level of exposure (delivery and read of messages) was not considered in this study. Aggregate 840 SMS and 420 minutes’ voice call were done to reach the intervention group.

The following SMS or Voice call was sent out to the study participants

**SMS**

Dear [first name] [middle name], this is a reminder for you to go to [name of the health center] for your (# of postnatal visit) postnatal checkup on date [date of the checkup date/month/year] please remember to visit the health center by (date of the week). Thank you!

**Voice Call**

“Hello, May I speak to [full name]. Hi [first name] I am calling you from [name of the health center] to remind you to attend your [# of postnatal visit] postnatal checkup on [date of the checkup date/month/year]. Please remember to visit the health center by (date of the week). Thank you!

Individuals in the control group received the usual care: appointment given by health professionals at discharge from the ward after delivery. In the Ethiopian health-care system, the health professionals working in the maternity ward are responsible for giving appointments to the mothers and no subsequent reminders were given.

### Outcome of the study

The primary outcome of this study was postnatal care visit compliance categorized on a three-level ordinal scale. The classifications were (1) no compliers, for mothers who never attended PNC, (2) partial compliers, for women who visited 1 or 2 times for their PNC and (3) full compliers, for women who attended the health facility 3 or more times during their PNC.

### Measurements

Selected demographic characteristics of the mother; age, maternal educational-level categorized into (no formal education, primary, secondary and higher education), marital status, husband’s education level and occupation status were included independent variables.

Maternal service utilization during the index pregnancy and the last child was assessed. The adequacy of ANC service was described as compliance to the recommended routine ANC services. Four or more ANC services attendance during the index pregnancy were considered as optimal ANC attendance, otherwise the participants were considered as having suboptimal ANC attendance. The past PNC experience was measured based on whether the mother had previously sought postnatal care for the senior siblings of the current baby, where mother was considered as a full compliant if she attended 3 or more postnatal visits, partially compliant, if the mother sought service 1 or 2 times and not compliant, if the mother never got PNC service for the last child. Parity was defined as the number of children ever delivered by a mother.

### Data management and analysis

The baseline data collected from the study participants were entered into Epi Data version 4.2.0 and the data were cleaned and exported to STATA version 13 (Stata Corp, College Station, TX) for analysis. Comparisons between the groups were made using Chi square test.

The primary outcome, postnatal visit compliance, was assessed for all assigned health centers. Outcome was fitted using a two-level bivariate and multivariate ordinal logistic regression model allowing for health centers clustering. The model was adjusted for study groups, selected sociodemographic characteristics (highest level of maternal educational attainment, husband educational status), level of compliance with PNC visit for the last child, ANC visits for the index pregnancy, parity and pregnancy desirability, decision making for medical care and perceived distance from health facility. Variables found to be significant during the two-level bivariate analysis were further analyzed using the two-level multivariate ordinal regression model.

The model was built under the assumption of individuals (Level I) were nested within health centers (Level II). The first model, null model (intercept only model), was fitted without independent variables. The empty model was used to determine the overall difference between the health centers and individuals in PNC compliance. The second model assessed the fixed effect and random effect of the model where individuals and the clustering level health center fitted in to the ordinal multilevel regression model.

In multilevel models, fixed effects referred to the average relation of individual variables on postnatal compliance and were expressed as adjusted odds ratio (AOR) and 95% confidence intervals. The random effects were the measure of variation in PNC visit compliance across the assigned health centers. We used the variance and intracluster correlational coefficient (ICC) to explain the variation. The ICC was calculated to see whether the variation in postnatal clinic visit compliance was primarily within or between the health centers.

### Ethics and consent

Ethical clearances for the study were obtained from the University of Ibadan/University College Hospital Institutional Review Board (UI/UCH/17/0050) and Ethiopian Public Health Institute Scientific and Ethical Review Committee (EPHI-IRB-034–2017). The permission to conduct the study was given by Addis Ababa health bureau. Written informed consent was obtained from mothers participating in the study after counseling. Parental or guardian informed assent was obtained for study participants less than 18 years of age. Women who refused to give consent to participate in the study were excluded without prejudice to their hospital care. Information gathered from the participants was stored in a secured cabinet by the first author and the contacts provided were confidentially kept by the research team.

## Results

### Baseline sociodemographic characteristics

At baseline, 350 (175 in the intervention and 175 in the control) women were enrolled from 16 health centers. The selected sociodemographic characteristics of study groups were similar– were shown in Table 1. The mean age at the baseline was 25.7(±5.3(SD)) years in the intervention group and 26.1(±5.6(SD)) years in the control group (p = 0.51). About 35% in the control group and 32% in the intervention group attended primary education. Majority 133 (76%) in the intervention group and 112 (64%) in the control group were married. More than half of the mothers in both groups decided by themselves to seek for medical care.

**Table 1.** Baseline socioeconomic and demographic characteristics of the study groups.

Characteristics	Intervention n = 175	Control n = 175	Overall N = 350	p-value
	n (%)	n (%)	n (%)	
**Age (**Mean ± SD (yrs.))	25.7 ± 5.3	26.1 ± 5.6	25.9 ± 5.4	0.51^a^
**Educational level**				
No Formal Education	40(22.9)	33(18.9)	73(20.9)	0.60
Primary Education	61(34.8)	56(32.0)	117(33.4)	
Secondary Education	47(26.8)	53(30.3)	100(28.5)	
Higher Education	27(15.4)	33(18.9)	60(17.1)	
**Occupational status**				
Civil Servant	41(23.4)	40(22.9)	81(23.1)	0.48
Self Employed	31(17.7)	37(21.1)	68(19.4)	
Student	81(46.3)	69(39.4)	150(42.8)	
Daily Laborer	22(12.6)	29(16.6)	51(14.6)	
**Marital status**				
Single	32(18.3)	53(30.3)	85(24.3)	0.07
Married	133(76.0)	112(64.0)	245(70)	
Widowed	10(5.7)	10(5.7)	20(5.7)	
**Husband educational status (n = 133)**				
No formal Education	9(6.8)	9(8.1)	18(7.3)	0.80
Primary education	21(15.8)	16(14.3)	37(15.1)	
Secondary education	37(27.8)	27(24.0)	64(26.1)	
Higher education	66(49.6)	60(53.7)	126(51.5)	
**Husband occupational status (n = 133)**				
Civil Servant	55(41.4)	49(43.8)	104(42.4)	0.73
House wife	27(20.3)	23(20.5)	50(20.4)	
Daily Laborer	27(20.3)	26(23.2)	53(21.6)	
Student	24(18.0)	14(12.5)	38(15.5)	
**Ethnicity**				
Amhara	80(45.7)	87(49.7)	167(47.7)	0.69
Oromo	46(26.3)	46(26.3)	92(26.3)	
Tigre	16(9.1)	17(9.7)	33(9.4)	
Gurage	33(18.9)	25(14.3)	58(16.6)	
**Religion**				
Christianity	127(72.6)	128(73.1)	255(72.9)	0.55
Islam	46(26.3)	43(24.6)	89(25.4)	
African traditional religion	2(1.1)	4(2.3)	6(1.7)	
**Family Size**				
1–2	23(13.2)	22(12.6)	45(12.8)	0.61
3–5	125(71.4)	119(68.0)	244(70.0)	
>5	27(15.4)	34(19.4)	61(17.2)	
**Decision maker at the household level**				
Self	94(53.7)	85(48.6)	179(51)	0.31
Husband	59(33.7)	58(33.1)	117(33)	
Other Family Member`	22(12.6)	32(18.3)	54(15.4)	
**Perceived distance to health facility**				
Big problem	59(33.7)	64(36.6)	123(35.1)	0.53
Not a big problem	116 (66.3)	111(63.4)	227(64.9)	
**Parity**				
1	80(45.7)	78(44.6)	158(45)	0.971
2–4	77(44.0)	78(44.6)	155(44.3)	
≥5	18(10.3)	19(10.8)	37(10.6)	

aindependent t test, p-value of the Pearson’s chi square test.

Table 2 presents the maternal health-care service utilization for the last and index pregnancy among the intervention and the control groups. There was no significant difference between the intervention and control group on past postnatal clinic attendance (p = 0.55). More than 70% in both groups had no PNC visits for the last child; 124(70%) of 175 mothers in the intervention group and 112(64%) of the 175 mothers in the control group attended 4 or more ANC visits during the index pregnancy. At baseline there was no statistically significant difference between the study groups’ reproductive and obstetrics-related characteristics.

**Table 2.** Baseline maternal health-care services utilization by women in the intervention and control groups.

Maternal health-care utilization	Intervention n = 175	Control n = 175	Overall N = 350	p-value
	n (%)	n (%)	n (%)	
**Had postnatal care for the last child**				
Yes	47(26.9)	52(29.7)	99(28.3)	0.55
No	128(73.1)	123(70.3)	251(71.1)	
**Level of compliance with PNC visits for the last child**				
No PNC	130(74.3)	125(71.4)	255(72.9)	0.77
Partial PNC	27(15.4)	32(18.3)	59(16.9)	
Full PNC	18(10.3)	18(10.2)	36(10.3)	
**Qualification of past postnatal attendants**				
Medical Doctor	3(6.4)	6(12.0)	9(9.3)	0.71
Health officer	8(17.02)	9(18.0)	17(17.5)	
Midwife	29(61.7)	26(52.0)	55(56.7)	
Nurse	7(14.9)	9(18.0)	16(16.5)	
**Number of ANC visit for the index pregnancy**				
<4 ANC visit	51(29.1)	63(36.0)	114(32.6)	0.171
4+ ANC Visit	124(70.9)	112(64.0)	236(67.4)	
**The index pregnancy was wanted**				
Yes	114(65.1)	113(64.6)	227(64.9)	0.911
No	61(34.9)	62(35.4)	123(35.1)	

p-value of the Pearson’s chi square test.

### Intervention phase

Complete primary outcome data was collected for 173(98.9%) of the mothers in the intervention group and 169 (96.5%) of the mothers in the control group; these were included for the final analysis. Figure 2 summarizes the postnatal visit compliance among the study groups. It indicates the attendance rate among the two groups, the confidence interval and the significance. Among mothers in the intervention group 92 (27%) partially and 16 (5%) fully complied with the recommended postnatal visits while, 66 (19%) and 10 (3%) in the control group partially and fully complied with the recommended PNC visits. There was a significant difference in PNC visit compliance among the study groups (p = 0.005).

Table 3 shows the binary ordinal logistic regression analyses (unadjusted odds ratios and 95% CI) for the association between intervention status, occupation status, educational level, frequency of ANC visits during the index pregnancy, parity, desire for the current pregnancy, decision making process, compliance of postnatal visits for the last child, perceived distance from the health center and PNC visit compliance during the bivariate analysis. The odds of complying with the PNC appointments were almost two times higher among respondents who received the mobile phone appointment reminders (SMS & Voice) compared to those who received the standard care crude odds ratio (COR: 2.03, 95% CI 1.34–3.08). Apart from this the odds of PNC visit compliance were three times higher among women who have attended higher education and above compared to those with no formal education (COR: 3.56, 95% CI 1.7–7.38). The postnatal care compliance was 5 times higher among civil servants (COR:5.68, 95% CI 2.85–11.28) compared to those who were self-employed. Mothers who fully complied with their PNC appointment in the past were 13 times more likely to comply with to the current postnatal care visit (COR:13.8, 95% CI 6.2–30.8).

**Table 3.** bivariate ordinal logistic regression analysis for association between postnatal care compliance and explanatory factors.

	PNC visit compliance			
Characteristics	No PNC n (%)	Partial PNC n (%)	Full PNC n (%)	COR (95% CI)
**Study group**				
Intervention	65(19)	92(27)	16(4.6)	2.03(1.34,3.08) *
Control	93(27.2)	66(19.3)	10(3)	ref
**Occupation**				
Civil Servant	17(5)	50(14.6)	13(3.8)	5.68(2.85,11.28) *
Student	76(22.2)	63(18.4)	11(3.2)	1.54(0.85,2.82)
Daily Laborer	29(8.5)	18(5.3)	1(0.3)	0.95(0.44,2.05)
Self Employed	36(10.5)	27(7.9)	1(0.3)	ref
**Educational level**				
No Formal Education	31(9)	38(11.1)	3(0.88)	ref
Primary education	62(18)	51(14.9)	1(0.3)	0.54(0.29,0.99) *
Secondary education	47(13.7)	42(12.3)	8(2.34)	0.81(0.43,1.51)
Higher education	18(5.3)	27(5.3)	14(4.09)	3.56(1.71,7.38) *
**Husband’s educational status (n = 133)**				
No Formal Education	8(3.1)	12(4.6)	2(0.77)	ref
Primary education	25(9.6)	15(5.8)	1(0.4)	0.20(0.06,1.63) *
Secondary education	51(19.7)	15(5.8)	1(0.4)	0.12(0.04,0.36) *
Higher education	39(15)	69(26.6)	21(8.1)	1.42(0.57,3.55)
**Husband’s occupational status (n = 133)**				
Civil Servant	36(14)	61(23.5)	14(5.4)	1.52(0.69,3.37)
Self Employed	37(14.3)	15(5.8)	1(0.4)	0.28(0.17,0.71) *
Daily Laborer	33(33)	20(7.7)	2(0.8)	0.45(0.18,1.08)
Student	17(6.5)	15(5.8)	8(3.1)	ref
**PNC visits compliance for the last child**				
No PNC	137(40.1)	98(28.6)	12(3.5)	ref
Partial PNC	16(4.7)	41(12)	2(0.6)	3.79(2.06,6.98) *
Full PNC	5(1.5)	19(5.5)	12(3.5)	13.86(6.23,30.8) *
**Number of ANC visit for the index child**				
<4 ANC	72(21)	35(10.2)	1(0.3)	ref
4+ ANC	86(25)	123(35.9)	25(7.3)	3.55(2.17,5.79) *
**Parity**				
1–2	112(32.7)	104(30.4)	15(4.4)	ref
3–4	28(8.2)	37(10.8)	10(3)	1.74(1.09,2.71) *
≥5	18(5.2)	17(5)	1(0.3)	1.23(0.61,2.65) *
**Sex of the newborn**				
Male	84(24.5)	80(23.4)	14(4.1)	ref
Female	74(21.6)	78(22.8)	12(3.5)	1.02(0.67,1.55)
**Current pregnancy wanted**				
Yes	95(27.8)	108(31.6)	24(7)	1.99(1.27,3.12) *
No	63(18.4)	50(14.6)	2(0.6)	ref
**Perceived distance to health facility**				
Big problem	28(8.2)	12(3.5)	2(0.6)	ref
Not a big problem	130(38)	146(46.2)	24(7.1)	2.37(1.16,4.87) *

p-value*: <0.05 COR; crude odds ratio 95% CI: confidence interval (95%); ref: reference group.

Table 4 presents the multilevel ordinal logistic regression showing association of compliance with the recommended PNC visits between the two study groups (p < 0.001) controlling for highest educational level, husband educational status, level of compliance with PNC visit for the last child, number of ANC visit, parity and pregnancy desirability, decision making for medical care, perceived distance from health facility and moreover, for the cluster level variations. There was no significant variability in the odds of postnatal visit compliance between the health centers. The intracluster correlation coefficient (ICC) was 8.9%.

**Table 4.** Multilevel model, random effect size and variability among the cluster factors and postnatal compliance.

	PNC visit compliance	
Characteristics	Model I^a^ AOR (95% CI)	Model II^b^ AOR (95% CI)
**Group**		
Intervention		2.98(1.51,5.81) *
Control		ref
**Occupation**		
Civil Servant		1.6(1.02,6.24) *
Student		0.64(0.34,2.38)
Daily Laborer		2.42(0.42,12.18)
Self Employed		ref
**Highest Level Educational Attainment**		
No Formal Education		ref
Primary education		1.31(0.51,3.33)
Secondary education		1.04(0.36,3.01)
Higher education		1.921(0.63,5.83)
**Husband Educational Status**		
No Formal Education		ref
Primary education		0.06(0.01,0.35) *
Secondary education		0.03(0.01,0.12) *
Higher education		1.73(0.38,7.79)
**Level of compliance with PNC visits for the last child**		
No PNC		ref
Partial PNC (1–2)		2.90(1.08,7.75) *
Full PNC		3.29(1.55,10.3) *
**Number of ANC visit**		
<4 ANC		ref
4+ ANC		1.54(1.09,2.59) *
**Parity**		
1–2		1.44(1.09,21.40) *
3–4		1.47(0.41,21.40)
≥5		ref
**Current Pregnancy Wanted**		
Yes		2.23(0.59,8.41) *
No		ref
**Decision making**		
Self		2.34(1.25,4.59) *
Husband		1.36(0.68,2.71)
Other relatives and families		ref
**Perceived distance to health facility**		
Big problem		ref
Not a big problem		1.25(0.38,4.11)
**Random effect parameters**	**Model I**	**Model II**
Community level Variance (SE)	0.324 (0.06, 2.03) (18.2%)	0.79(0.23,0.89) (50%)
ICC (%)	8.9%	19.4%
Model fit statistics		
Log-likelihood	−310.5	−164.36
AIC	627	378

Note: ^a^ Empty model which contains no explanatory variables (intercept only model) but partition the variance into two component parts, ^b^ Model II: model which contains individual level variables equated by cluster level variable (health centers) AIC = Akaike information criterion, CI = confidence interval, ref = reference category, SE = standard error, ICC = intracluster correlation coefficient.

Log-likelihood = this is the log-likelihood of the fitted model. The value of the log likelihood (−164.36) in model II indicates a better fit

*: significant at 5% level of significance.

The odds of attending PNC was two times higher among the intervention group compared to the group the control group (AOR: 2.98, 95% CI 1.51–5.81). Mothers who received 4 or more ANC service were 1.5 times more likely to comply with the recommended PNC services (AOR:1.54, 95% CI 1.09–2.59). Similarly, mothers who decided to seek care by themselves were 2 times more likely to comply with postnatal care (AOR:2.34,95% CI 1.25–4.59).

Table 5 shows whether the SMS reminders helped mothers to attend postnatal clinic or if they recommend to others. More than 90% of the mothers reported the reminders helped them to attend postnatal clinics, while 6.5% of them did not. The majority more than 90% of the respondents recommended the reminders to their family member and or friends or relatives.

**Table 5.** Acceptability of the reminders for postnatal appointment[n = 124].

Acceptability	n	%
**The reminder was helpful to visit health center during your postnatal**		
Helpful	114	91.9
Not helpful	8	6.45
Not sure	2	1.6
**Recommend reminders to family member or friend**		
Yes	113	91.1
Not sure	11	8.9
Total	124	100

## Discussion

The goal of this study was to evaluate the effect of enhanced (SMS/Voice call) reminders and factors associated with postnatal visit compliance among women in Addis Ababa, Ethiopia.

There is an ample of evidence and recommendations from the WHO to support the use mHealth as a tool to improve the quality of health care [36–38]. The fast-growing coverage and high penetration of wireless technologies in developing countries is seen as an opportunity to be integrated for different health services, health outcomes and interventions to improve the quality of health care [16].

We observed a significant association between study groups, mother’s occupation, husband educational status, having optimal ANC attendance during the index pregnancy, the desire to have the current baby, parity and postnatal clinic visit compliance.

We found higher odds of postnatal compliance with the recommended PNC visits among the intervention group compared to the control group. This finding was in line with other prior randomized and nonrandomized studies which consistently reported that electronic reminders significantly improved patients adherence to different health service appointments like maternal health services, outpatient and other health-care units [22,23,40–47]. Therefore, it can be deduced that mobile reminders and other integrated enablers are effective in improving the health-care service attendance by modifying the attitude of the patients [48]. Odeny et al [49], in a study on the use of mHealth in postpartum care of mothers with HIV showed that simple reminders which provide details of timing and location of appointments are effective, in helping a forgetful patient to attend their appointment. The content of the message, the type of behavior being reminded, literacy level, the cost incurred and other contextual factors might have influenced the effect of reminder demonstrated in this study. Considering the effectiveness of the mHealth in improving the maternal health-care utilization, we recommend the ministry of health to consider mobile reminders as a routine practice to improve one of the neglected maternal health-care service in the maternal continuum of care (PNC).

This study also revealed a higher acceptance of reminders among the mothers in the intervention group. More than, 90% of the mothers were positive about getting reminders for their PNC appointment. This finding was in line with several studies done in other places [40,50–53]. This finding was higher compared to study done in Ilorin Nigeria, which showed 69% acceptance of reminders for immunization service [54]. The variations in these two reports might be due to differences in culture and mobile phone coverage.

We also found that, compliance with ANC clinic attendance in the index pregnancy (having 4 or more ANC visits) were found to increase the compliance with the PNC visits. Similar studies from Ethiopia, Ghana and Indonesia supported this finding [9,55–57]. The positive health-seeking behavior among those with higher ANC attendance might be the reason. The ANC is one of the first contact during pregnancy and important component in the maternal continuum of care. It can be considered as window of opportunity for health professionals to give health education and to raise the health awareness of the respondents. However, this assertion has been disputed by reports from studies elsewhere that failed to establish a significant relationship between number of ANC visits and PNC visit compliance [11,58]. Difference in study design and cost incurred to maternal services in different countries may explain this variation. Certainly, laying more emphasis on improving ANC attendance and the quality of the ANC should be one of the strategies to encourage the postnatal visit compliance in developing countries.

Decision making autonomy was also found to be one of the determinants for PNC visit compliance in this study. Mother’s decision to attend medical care was found to be positively associated with higher odds of PNC visit compliance. Mothers who decide by themselves to seek medical care were two times more likely to comply with PNC appointments. This finding was in line with studies from Ethiopia, Bangladesh, and a systematic review which showed having higher decision making autonomy was associated with higher medical service utilization [10,59,60]. Adhikari [61], in Nepal also documented low level of women’s autonomy was associated with low maternal service utilization. This consistent findings among studies could be explained by the fact that decision-making is always accompanied by enabling factors like education and empowerment which facilitates higher health-care attendance [62]. It is therefore necessary that women with lower education should be targeted for reminders and health education in order to enhance PNC visits by them.

### Strength and limitations of the study

This study used a rigorous methodology and statistical analysis which considered the variation within individual variables and between the health centers. However, there were few limitations. The data on acceptability were not supported with robust qualitative technique. The final analysis did not consider level of exposure among intervention group, number of reminders received and read. This study has not addressed the outcome of attending postnatal services among the study groups. We recommend further rigorous studies which covers cost-effectiveness analysis incorporating the morbidity and mortality averted due to the intervention and moreover attendance of the maternal service.

## Conclusion

Dropout from maternal continuum of care leads to lost opportunity and low-service quality. In this paper we found, mobile phone SMS reminders are likely to improve PNC visit compliance and are acceptable approaches to remind the client. And also, optimal ANC attendance, past PNC compliance, women autonomy on decision making, and desire for pregnancy was significantly associated with PNC compliance. Further studies addressing the cost-effectiveness analysis and users experience using qualitative approach is recommended. Moreover, Ministry of Health could integrate mobile phone reminders in the routine postnatal follow up package.

## Paper context

High PNC compliance will avert the toll maternal and neonatal death. There is an indication from WHO and systematic reviews, that mobile phone reminders can be used to improve attendance to health appointments. However, there is no rigorous study evaluated the role of this reminders in postnatal care service in the current study setting. We hope our study will provide a basis for further studies and policy translation.
